# “We are nurses – what can we say?”: power asymmetries and Auxiliary Nurse Midwives in an Indian state

**DOI:** 10.1080/26410397.2022.2031598

**Published:** 2022-02-16

**Authors:** Priya Das, Sudha Ramani, Tom Newton-Lewis, Phalasha Nagpal, Karima Khalil, Dipanwita Gharai, Shamayita Das, Rochana Kammowanee

**Affiliations:** a Consultant, Oxford Policy Management Limited, New Delhi, India. *Correspondence*: priya.das@opml.co.uk; b Senior Consultant, Oxford Policy Management Limited, New Delhi, India; c Freelance Consultant, Newland, Witney, UK; d Assistant Consultant, Oxford Policy Management Limited, New Delhi, India; e Nurse Researcher, Oxford Policy Management Limited, New Delhi, India; f Research Assistant, Oxford Policy Management Limited, New Delhi, India

**Keywords:** India, auxiliary nurses and midwives, health systems strengthening, gender, power, empowerment, ethnography

## Abstract

In India, nurses and midwives are key to the provision of public sexual and reproductive health services. Research on impediments to their performance has primarily focused on their individual capability and systemic resource constraints. Despite emerging evidence on gender-based discrimination and low professional acceptance faced by these cadres, little has been done to link these constraints to power asymmetries within the health system. We analysed data from an ethnography conducted in two primary healthcare facilities in an eastern state in India, using Veneklasen and Miller’s expressions of power framework, to explore how power and gender asymmetries constrain performance and quality of care provided by Auxiliary Nurse Midwives (ANMs). We find that ANMs’ low position within the official hierarchy allows managers and doctors to exercise “power over” them, severely curtailing their expression of all other forms of power. Disempowerment of ANMs occurs at multiple levels in interlinked and interdependent ways. Our findings contribute to the empirical evidence, advancing the understanding of gender as a structurally embedded dimension of power. We illustrate how the weak positioning of ANMs reflects their lack of representation in policymaking positions, a virtual absence of gender-sensitive policies, and ultimately organisational power structures embedded in patriarchy. By deepening the understanding of empowerment, the paper suggests implementable pathways to empower ANMs for improved performance. This requires addressing entrenched gender inequities through structural and organisational changes that realign power relations, facilitate more collaborative ways of exercising power, and create the antecedents to individual empowerment.

## Résumé

En Inde, les infirmières et les sages-femmes sont essentielles pour la prestation des services publics de santé sexuelle et reproductive. La recherche sur les facteurs contrariant leur activité s’est principalement centrée sur leur capacité individuelle et les restrictions systémiques de ressources. En dépit de données émergentes sur la discrimination sexuelle et la faible acceptation professionnelle auxquelles font face ces catégories, peu de mesures ont été prises pour lier ces contraintes aux asymétries de pouvoir qui existent au sein du système de santé. Nous avons analysé les données d’une étude ethnographique menée dans deux centres de soins de santé primaires dans un État de l’Inde orientale, en utilisant le cadre des expressions de pouvoir de Veneklasen et Miller, afin d’étudier comment le pouvoir et les asymétries entre hommes et femmes limitent la performance et la qualité des soins assurés par les infirmières sages-femmes auxiliaires. Nous avons constaté que la médiocre position de ces femmes dans la hiérarchie officielle permet aux administrateurs et aux médecins d’exercer un « pouvoir sur » elles, réduisant gravement leur expression de toute autre forme de pouvoir. La privation de pouvoir des infirmières sages-femmes auxiliaires se produit à de multiples niveaux, de manières liées et interdépendantes. Nos résultats contribuent aux données empiriques, faisant progresser la compréhension du genre comme dimension structurellement enracinée de pouvoir. Nous montrons comment le faible positionnement des infirmières sages-femmes auxiliaires reflète leur manque de représentation dans les postes de définition des politiques, une absence virtuelle de politiques sensibles aux différences entre hommes et femmes, et, en fin de compte, des structures de pouvoir ancrées dans le patriarcat. En approfondissant la compréhension de l’autonomisation, l’article suggère des voies réalisables pour responsabiliser les infirmières sages-femmes auxiliaires afin d’améliorer leur performance. Cela exige de s’attaquer aux inégalités de genre profondément enracinées avec des changements structurels et organisationnels qui réalignent les relations de pouvoir, facilitent des façons plus collaboratives d’exercer le pouvoir et créent les antécédents de l’autonomisation individuelle.

## Resumen

En India, las enfermeras y parteras desempeñan un papel fundamental en la prestación de servicios de salud sexual y reproductiva del sector público. Las investigaciones sobre los impedimentos a su desempeño laboral se han enfocado principalmente en su capacidad individual y en las restricciones sistémicas de recursos. A pesar de la evidencia emergente sobre la discriminación de género y la baja aceptación profesional enfrentada por estas categorías de personal de salud, no se ha hecho mucho por asociar estas restricciones con las asimetrías de poder en el sistema de salud. Analizamos los datos de una etnografía realizada en dos establecimientos de salud de primer nivel, en un estado oriental de India, utilizando el marco de expresiones de poder de Veneklasen y Miller, para explorar cómo las asimetrías de poder y género restringen el desempeño laboral y la calidad de los servicios proporcionados por Enfermeras-Obstétricas Auxiliares (EOA). Encontramos que la baja posición de las EOA en la jerarquía oficial les permite a los administradores y médicos ejercer “poder sobre” ellas, disminuyendo en gran medida su expresión de todas las demás formas de poder. El desempoderamiento de las EOA ocurre en múltiples niveles en maneras interrelacionadas e interdependientes. Nuestros hallazgos contribuyen a la evidencia empírica, lo cual mejora la comprensión del género como una dimensión de poder arraigada estructuralmente. Ilustramos cómo la posición débil de las EOA refleja su falta de representación en puestos de formulación de políticas, la ausencia virtual de políticas sensibles al género y, a la larga, estructuras de poder organizacional arraigadas en el patriarcado. Al profundizar la comprensión de empoderamiento, el artículo sugiere vías aplicables para empoderar a las EOA a mejorar su desempeño laboral. Para ello, es necesario abordar las desigualdades de género arraigadas por medio de cambios estructurales y organizacionales que realineen las relaciones de poder, faciliten maneras más colaborativas de ejercer poder y creen los antecedentes para el empoderamiento individual.

## Introduction

In India, Auxiliary Nurse Midwives (ANMs) are key providers of sexual, reproductive, maternal, neonatal, and child health services in the public health system.^1,2^ The ANM cadre was first created in India in the 1950s to focus on basic midwifery and child health.^1^ The scope of ANMs’ work has since expanded: currently, ANMs are responsible for a range of activities pertaining to family planning, immunisation, infection control, and other primary care activities.^1,3^ They are posted at public primary health facilities and also undertake outreach work alongside other community health worker cadres.^2^ In India, policy dictates that General Nurse Midwives (GNMs), who are more highly trained,* have primary responsibility for midwifery in public health facilities. However, in some low-resource states where there is a shortage of GNMs, ANMs also undertake additional clinical and midwifery roles.^3^ Currently, the 18 months of pre-service training that ANMs receive is focused mainly on their outreach work and does not equip them for midwifery roles.^3^

Concerns raised about ANMs’ performance are mostly attributed to gaps in individual capability (knowledge gaps and lack of continued professional training) and to systemic constraints (lack of supportive infrastructure, mentoring, and supervision).^4,5^ There is increasing evidence that these cadres face gender-based discrimination, frustration, low motivation, compromised professional acceptance, and feelings of helplessness.^6,7^ However, literature explicitly linking the above-mentioned constraints to health system power asymmetries, particularly how gender-based power relations affect everyday ANM performance, is lacking. This paper attempts to help fill this gap.

The need to study power within health systems explicitly – in order to improve and transform those systems – has often been expressed in recent literature.^8,9^ However, there is limited empirical evidence, particularly from low- and middle-income countries (LMICs), on how power relationships within health systems can affect service delivery.^10,11^ It is increasingly acknowledged that health systems entrench and reinforce existing gender norms and inequalities, with adverse impacts for the female workforce.^12–14^ Yet, how power is sourced from gender, or how gender is a power relation in itself, and the impact of this on providers and their performance, has been less researched.^15,16^ Only a few studies have highlighted issues related to gender-biased workforce policies, lack of protective measures for the female health workforce, and the absence of gender considerations in managerial, leadership, and accountability practices.^6,15,17^

In this paper, we offer empirical evidence to explore and aid further understanding of how gendered power relations constrain performance and quality of care. We do this by reporting evidence from an ethnographic study of ANMs in two primary healthcare facilities in an eastern state in India. We have made the decision not to name the state, which hereafter is referred to as Esma.

Esma is one of the most populous states in India; it has low income per capita and a mostly rural population.^18^ Esma has one of the lowest per capita health expenditures in India, and, like other low-resourced Indian states, its public health system is characterised by inadequate infrastructure, erratic supply of equipment, and a severe shortage of clinical and non-clinical staff.^19,20^ Despite recent improvements, health outcomes in the state remain poor.^18^ Due to a shortage of staff nurses, many ANMs become by default the main providers of childbirth and newborn care services at primary facilities.^21^ To enable ANMs to provide midwifery services, the Government of Esma, with the support of donors and development partners, provides ANMs with on-site, in-service nurse mentoring focused on improving basic and comprehensive emergency obstetric and newborn care. Despite these inputs, ANMs’ provision of quality birth and newborn care remains a concern. Quality of care studies in Esma, as in other states with similar mentoring programmes, show that a range of routine quality of care practices are not adhered to during childbirth (including triaging, checking for complete removal of the placenta, routine episiotomy, and resuscitating newborns).^22,23^

In this paper, we use the “expressions of power” framework^24^ to make explicit the interlinkages between power and ANMs’ performance. Using evidence, we argue that in order to improve ANMs’ performance, the power asymmetries that are entrenched in the health system and everyday functioning of health facilities need to be addressed.

## Methodology

### Approach and rationale

The data used in this paper are drawn from an ethnographic study conducted at two primary level health facilities within a larger evaluation of a health system strengthening intervention in Esma. The aim of the ethnographic study was to understand why nurses and managers did not implement practices despite having the knowledge to do so (the “know–do gap”). This included a focus on the health system’s “intangible software” – that is, the “ideas and interests, values and norms, and affinities and power that guide actions and underpin the relationships among system actors and elements” (p. 2).^13^ Intangible software operates as unwritten scripts that are enacted through hierarchies and power disparities within a health system. To make such unwritten scripts apparent, we chose to apply a focused ethnography approach. Such an approach is usually used to study contained phenomena within shorter time periods than apply in classical ethnographic approaches.^25^ The use of focused ethnography in health systems research has been found to be effective in capturing in-depth and nuanced data on the relationships between power, knowledge, and practice, making apparent what is otherwise difficult to discern.^25,26^

### Ethics

Ethical approval was granted in August 2019 (Institutional Review Board Number 10026/IRB/19-20) by Sigma Research and Consulting Private Limited. The research was conducted with the permission of Esma’s state government.

### Fieldwork and data collection

The selection of the research sites was carried out based on monthly birth load (i.e. the number of births per month), the range of services provided, and overall facility performance, and in consultation with the development partners who were implementing the ongoing health system strengthening initiative. After visits to 10 facilities, two sites were purposively selected. Both facilities are Community Health Centres established at the sub-district level and are typical of block-level Community Health Centres in Esma. We purposely chose two well-performing facilities (assessed in terms of the birth load and available services and infrastructure) so as to mitigate some of the more obvious reasons for prevalent know–do gaps, like poor infrastructure or the presence of ANMs that have not received inputs from the ongoing government mentoring initiative. Other selection criteria included the feasibility of fieldwork and the willingness of staff to take part in the study.

Data collection was undertaken by two research teams, both comprising a male and a female social scientist and a qualified nurse researcher: a total of six researchers in all. A social anthropologist and a public health physician led the study. Fieldwork was conducted between August 2019 and March 2020. Data were collected through five cycles of embedded fieldwork of three to five weeks’ duration each, with research teams spending six to eight hours daily at the facilities, five days a week. Debriefing, initial analysis, knowledge-building, reflection, and planning by the teams took place between field visits, allowing each cycle to build on insights from preceding cycles.

To ensure standardisation and inter-observer reliability, 24 semi-structured, open-ended tools were developed to capture all processes and practices related to facility functioning, staff interactions, and specific reproductive, maternal, and newborn child health clinical and managerial practices. In addition to structured formal interviews, the teams engaged in unstructured conversations and real-time participant observation in the setting but not in the activities. In addition, the teams used shadowing, i.e. following and observing individual stakeholders to learn about their routines and typical forms of engagement with their activities and other stakeholders.^27^ A summary of the methods and data collection is presented in Table 1.

**Table 1.** Data collection tools and methods used

Type of qualitative method	Respondents interviewed/events observed	Number of respondents	
		Facility A	Facility B
**Interviews**			
Structured (formal) and unstructured (informal) interviews	Medical Officers and Clinical Managers in-Charge (CMICs), hospital managers, and clinical and non-clinical staff	86	75
**Observations**			
Direct observation of births	GNMs, ANMs, and other birth assistants	35	15
Shadowing	Clinical and non-clinical staff	13	4
Observation of referral procedures	Birth asphyxia, larger than usual baby, short stature of mother	5	7
Observation of respectful maternity care (how the GNMs and ANMs dealt with the women in providing birth-related services, including quality of care)	Clinical providers of birthing, antenatal, and postnatal care	5	3
Observation of critical events	Birth asphyxia, genetic disorder, postpartum haemorrhage, uterine prolapse, cervical tear, congenital malformation of cervix, discharge against medical advice	8	11
Observation of facility-wide services or events	Official inspection and monitoring visits and meetings, national health programmes, scheme-based antenatal care days, immunisation days, and family planning operation days	9	2
Observation of meetings	Weekly meetings of ANMs and outreach workers, weekly and monthly meetings related to quality improvement, and weekly clinical discussions between nurses and management	8	6
**Mapping**	Graphical representation and description of facilities		

Data collection was open-ended, iterative, and flexible, in order to capture all dimensions of interest. This allowed the triangulation of information and the construction of in-depth insights over time. Interviews were conducted in Hindi and were recorded with permission. Detailed notes were taken where consent to record interviews was not given. Formal verbal consent to participate in the study, including being interviewed and observed, was received from all providers and managers, using an informed consent form. Formal verbal consent (using informed consent forms) with regard to observing critical events and provider practices was also received from individual birthing women and their accompanying family member. The research teams received approval from the ethics review board to observe birth-related care, including antenatal care, birthing care, and postnatal care.

Nurse researchers in the teams had long experience of working in Esma. Thus, the research teams were aware that in observing birthing they would witness poor quality labour room practices that could be potentially dangerous (for example, failure to sterilise equipment, unindicated use of uterotonics to augment labour). However, given that the nurse researchers were non-practitioners, from a research ethics perspective we were advised by the ethics approval committee to restrict our engagement only to non-participant observations. Further, most of the poor-quality practices observed have been well documented in the context of Esma and are known to health authorities at all levels. We have presented our findings to the state authorities, while anonymising the sources of the data and observations.

The researchers maintained expanded field notes with thick descriptions to capture the contexts of what they were observing and hearing, and collated these thematically using the data collection tools. Team briefings were held at the end of each day to discuss emerging issues. The fieldwork was conducted primarily by the field teams. The co-leads of the study visited both sites twice during the data collection period to spend a few days in each facility and to engage with the field teams in real time.

### Data analysis

Data analysis was inductive, using a grounded theory approach, and was iterative in nature, with preliminary analysis conducted in the intervals between data collection cycles. As a focused ethnography, the broad enquiry domains were identified *a priori*. The analytical themes within each domain were drawn and analysed using the content analysis method,^28^ using interview transcripts, field diaries, and observation notes. Data were thematically codified and tabulated manually using Excel and Word to identify emerging patterns and to arrive at core analytical constructs iteratively. Preliminary interpretations were collectively discussed within the research teams and continuously challenged for reflexivity and for robustness. The analysis was situated within the social, political, and historical context of which facility staff were a part and was informed by reviewing relevant literature related to Esma and its health system, and to theoretical frameworks related to performance accountability, gender, power, corruption, and other aspects. All data were processed by the research teams.

In the initial inductive analysis both power and gender emerged as strong underpinnings of the *de facto* practices, and the focus of themes was sharpened in the successive cycles, including mapping the sources and typology of power.^9^ Given this, we chose to deepen the analysis using the “expressions of power” framework detailed in the next section as it most aptly captured the relationship between the ANMs, as the prime providers of services, and the management.

### The decision to not name the state

Our study was conducted in the context of a donor-funded health system strengthening intervention supporting Esma’s state government. Our role was to support both the donor and the government to learn from the health system strengthening intervention. While we had government approval and support to undertake the ethnographic study, given the political sensitivities involved and the need to maintain/protect author–funder–government relationships, we took the decision not to name the state. Since the ethnography yielded rich data on lesser-known aspects that impact provider performance, especially ANMs (a cadre that operates throughout India), we decided to disseminate the findings without naming the state.

### The conceptual framework: “expressions of power”

The analysis in this paper is structured around the “expressions of power” framework,^24^ which is outlined in Table 2. Power here is perceived as a dynamic and multidimensional concept, with four expressions: power over, power to, power with, and power within. These four expressions of power are interconnected, and notions of empowerment in this framework are conceptualised as a shifting away from coercive “power over” relationships to more collaborative ways of working.^24^ This framework belongs to a broader set of rights-based power analysis approaches.^29,30^ In this set of approaches, “empowerment” is considered as tackling the disadvantages that stem from the ways in which relationships of power influence choices, opportunities, and behaviours.^29^ Further, the theoretical frameworks derived from this set of approaches are intended to be practitioner-oriented and are meant to transform existing power relations to bring about empowerment.

**Table 2.** Expressions of power

Type of power relation	Description
Power over	Often associated with repression, abuse, and coercion. Here power is looked at as “zero sum”, wherein having power implies taking it from someone else. It is the “power over” certain groups that other groups have that leads to disempowerment and marginalisation.
Power to	Capacity to take action and change existing hierarchies. Emphasis on joint decision-making and action, and equitable relationships.
Power with	Finding common ground based on different interests, support, and solidarity. Emphasis on collective action and alliance-building.
Power within	A person’s sense of self-worth and knowledge. Emphasis on individual consciousness and awareness.

Source: VeneKlasen and Miller^24^

Power as a concept is complex and difficult to define.^9,24^ As used in this framework, power is defined as “the degree of control over material, human, intellectual and financial resources exercised by different sections of society. The control of these resources becomes a source of individual and social power” (p. 41).^24^ Underpinning the expressions of power framework is the understanding that power is relational and contextual – it is exercised in the social, economic, and political relations between individuals and groups.^31^ Thus, operating between actors within the health system, power is demonstrated through the closely related constructs of authority, accountability, intersectionality, and hierarchy, and is embedded in social, historical, political, and cultural contexts.^9^

## Findings

### The facility context

The power positioning of ANMs needs to be understood within their organisational context and working environment. The two rural primary-level Community Health Centres where the study was conducted – Facility A and Facility B – provide antenatal, birth, postpartum, and newborn care, immunisation, family planning, referral, ambulance services, and outpatient care. Facility B also serves as a first referral unit and has an operating theatre equipped for sterilisation (both male and female) and caesarean sections.

The service-readiness levels of these facilities, including human resources, infrastructure, drugs, supplies, and equipment, were found to be comparable to most primary-level facilities surveyed in the state: they are quite low and do not meet the requirements of the Maternal and Newborn Health Toolkit (MNHT) developed by the Ministry of Health and Family Welfare (MoHFW).^32^ Neither facility had the minimum human resources required to deliver basic emergency obstetric and newborn care according to the MNHT guidelines (see Table 3).†

**Table 3.** Birth caseload and availability of clinical staff in the facilities

	Facility A	Facility B	Human resource requirements for 200–500 births/month in the maternity wing, as per MNHT
Monthly birth caseload	300–500	350	
Medical doctors (MBBS)	1	4	5
AYUSH doctors	7	4	–
GNMs	–	3	9
ANMs	14	17	4
Obstetricians and gynaecologists	–	–	1

While the facilities’ birth load was high, both were short of doctors, GNMs, and ANMs. Even within the overall shortage, at least three GNMs or ANMs are needed in maternity wings, in each of three shifts, to take care of the labour room, antenatal and postnatal wards, registering, and triaging. However, during our fieldwork in the two facilities we found that usually only two providers (ANM and/or GNM) were available – and sometimes just one was left attending to three births. Frequent absenteeism has led to further “created” shortages. For example, at Facility A, 13 ANMs were on the roster during our fieldwork but only eight were working because two had been transferred and three rarely reported for duty. The shortage of doctors with a Bachelor of Medicine and Bachelor of Surgery (MBBS) degree was made up for through Ayurveda, Yoga, Unani, Siddha, and Homeopathy- (AYUSH-) qualified doctors.

In addition, we found that staff at the facilities came under two different supervisory structures: permanent staff of the state health establishment, who had secure tenure and better benefits; and contractual staff hired through a federal scheme, the National Health Mission, who had insecure tenure and lower pay than the establishment staff. These two supervisory structures further complicated existing organisational hierarchies.

Because of acute clinical staff shortages and involvement in multiple programmes, we found that ANMs worked across multiple and competing programme priorities within the same shift. In addition to these tasks, they were mandated to attend meetings and trainings. Further, maintaining updated documentation and records related to the labour room and other services significantly added to their work burden.

### ANMs and expressions of power

The findings on power have been organised and analysed according to the four expressions of power set out in VeneKlasen and Miller’s framework.^24^

#### Power over

Of the four expressions of power, “power over” ANMs was found to be the most predominant in the two facilities. Occupying the lowest rung of a hierarchically structured health system, ANMs were found to lack legitimate or positional power. Consequently, they were found to be formally accountable to a range of authorities above them – doctors, management administrators, implementation partners, and district authorities, most of whom were male. Their support structure within the system was found to be limited by the fact that positions for the supervisory female cadre in Esma, meant to oversee the performance and professional growth of nurses and midwives, have been vacant since their inception over a decade ago.

We found that expressions of “power over” were ingrained in the day-to-day interactions between ANMs and male management staff, often underpinned by gender-based biases and discrimination. For example, one of the clinical managers in Facility A insisted that ANMs’ performance suffers because they are “women”:

*“Being women, they lack the strong will and determination. If they had that kind of determination, like men do, they would have been able to perform despite the constraints.”* (CMIC, Facility A)

Further, in the same facility, we observed that spaces designated for ANMs were occupied by male management and clinical staff. We also learnt that ANMs had no rest room or access to clean toilets:

*“We use the toilet meant for the pregnant women or family … they are usually dirty. When rooms were being allotted, all the men occupied all the rooms first. Then, at the end, the women were allotted rooms. Our current ANM duty room is meant to be a ward. This is my home and my office. We don’t get the other rest rooms like we are supposed to.”* (ANM, Facility A)

We found instances where the designated working areas for ANMs were not suitable. For example, in Facility B the nursing station in the labour room lacked ventilation. Thus, ANMs resorted to sitting in the cooler triaging room because of the extreme heat, limiting observations of labour.

The gendered “power over” ANMs was also manifested in the lower levels of physical security available to them. We found that, in both facilities, security was prioritised for male management and clinical staff, with no security staff assigned to the maternity wing, despite repeated requests. ANMs faced consistent harassment, demands, and even threats from patients’ family members in relation to providing services that were not advisable (such as unindicated use of uterotonics to augment or speed up labour). They were also harassed if there were complications during birthing. One ANM shared her frustrations with the inadequate security:

*“They [security guards] only stay in the OPD [Outpatient Department] and emergency area. They come to the maternity section when there is a monitoring visit by the higher authorities. We have to deal with the male family members; you’ll often find us shouting at them in frustration – maybe using bad language … ”* (ANM, Facility B)

We observed that ANMs in the facilities often gave in to the demands of family members, due to fears of repercussions from the community. The lack of adequate security also meant they could not prevent male family members from entering and crowding the labour room floor at will.

The acute shortage of doctors, obstetricians, and gynaecologists in both the facilities meant that GNMs and ANMs (only ANMs in Facility A) were *de facto* the main service providers for sexual and reproductive health, maternal, and neonatal care. ANMs often bore the brunt of criticism for poor facility performance. We also observed instances where facility managers drew upon their formal power to compel ANMs to meet unrealistic targets. They served informal show-cause‡ notices (issued but not formally registered) and threatened to withhold ANMs’ salaries or increments. We also observed that management was often delivered in punitive ways, with public scolding, or authoritatively, using a raised voice. This occurred even though managers were aware of the infrastructural and workload constraints faced by the ANMs.

These numerous instances described above illustrate how the “power over” ANMs in the two facilities created a clear asymmetry between ANMs’ multiple responsibilities and their low level of formal, positional, and legitimate power, with detrimental effects on service provision.

#### Power to

ANMs’ “power to” exercise their agency, access resources, and voice their concerns in the two facilities was found to be undermined by the strong wielding of “power over” by managers. For instance, ANMs felt they must comply with decisions made by management. Formal, objective performance assessments – through the ANM Annual Performance Report – were often not undertaken. Informal assessment and opportunities for training and learning were found to be based on patronage and complicity in management malpractices related to corruption, further muting ANMs’ “power to” raise any objections.

We found that management staff expected that nurses would not defy them in any way, even if they were in the wrong, and that ANMs feared punitive actions and reprisals from the facility authorities. In Facility A we encountered an instance where a complaint was submitted by a ward boy against the Clinical Manager in Charge (CMIC). In this case, when they were compelled to conduct a formal process, district-level officials, who were keen to protect their own cadre, did not maintain confidentiality or privacy. All of the ANMs, who had several grievances regarding how the CMIC behaved with them, signed a declaration clearing the CMIC of any bad conduct. Reflecting on this powerlessness, an ANM stated:

*“They [the outside authorities] will go away – I still have to come back tomorrow and work under him [the facility-in-charge]. Besides, no facility-in-charge gets removed because of an ANM’s complaints. They [the management] all protect each other; we end up suffering and being the bad person.”* (ANM, Facility A)

In both facilities, the research teams often heard the common refrain “We are nurses – what can we say?” and even “Just because we are women any man can come in and say anything – even the ward boy shouts at us”. From our observations, we felt that the potential for collaborative space between ANMs and managers was marred by the absence of any process for reciprocal accountability. We found that ANMs had no formal space or mechanism with which to hold medical officers and management accountable for their lack of clinical or managerial support. We observed multiple occasions when the designated doctors refrained from providing requisite clinical inputs, were absent during their shifts, and refrained from standing up for ANMs when family members of patients made unreasonable demands for antibiotics, uterotonics, or glucose.

We found only very limited ways in which ANMs could exercise their agency with respect to clinical decisions in the health facilities. We observed several instances where necessary equipment was lacking and where ANMs were told to “manage somehow”. We also observed instances where ANMs were compelled to take on clinical responsibility for complications during birth that they were not trained to take on. As one of the ANMs shared (with a sense of frustration and in a complaining tone):

*“We are supposed to do everything here when it comes to delivery. This [facility] is expected to have a lady obstetrician, but we do not have even an MBBS doctor. The doctors who are here have no knowledge. They say ‘Sister, you know better than me. Do what you think is right, don’t ask me’.”* (ANM, Facility A)

We also found instances where ANMs were restrained from making service-related decisions that they felt were within their capacity. For example, we observed that sometimes ANMs had been forced by managers to refer patients, despite them feeling confident that they could handle the case, particularly to meet referral targets set for the facility.

We also observed low levels of the “power to” expression in the way ANMs’ responsibilities, shifts, and rosters were managed. For instance, our research teams trailed the roster-making process and found that the nurse shift rosters were based on doctors’ desire to prioritise their private practice. One of the ANMs shared:

*“I am supposed to manage the roster for the nurses, but it is made according to the whims and fancies of the CMICs and Health Manager.”* (ANM, Facility A)

#### Power with

“Power with” refers to fostering collaborative relationships between ANMs and other facility staff – relationships that are built on mutual respect, trust, and solidarity. However, our observations suggested that such relationships were lacking in the two facilities. For example, ANMs and nurses are formally required to be part of facility quality improvement platforms and district quality control committees. However, we observed that, despite being designated as in charge of the labour rooms, ANMs’ participation in meetings was largely limited to providing work status reports and submitting requests for labour room supplies, equipment, and drugs. Observations of these meetings suggested that the management did not approve of ANMs voluntarily voicing issues that reflected negatively on their practices. For instance, at Facility A the available stethoscopes had been appropriated by a higher-order clinical staff member, who also asked for the single labour room stethoscope to be used in the outpatient department. In a quality assurance committee meeting, when the concerned ANM tried to raise the issue, she was told to “keep quiet, not talk nonsense, and focus on the more serious matters”.

In facilities where both ANMs and GNMs were present, we observed the “power with” dynamics to be even more complex. Many issues affect the power hierarchies between ANMs and GNMs, including the nature of the employment contract (contractual vs permanent), the nature of the work (outreach vs facility-level), training differentials, and religious and ethnic identity (Christian and non-Christian; tribal and non-tribal). Preferential treatment and patronage of some ANMs and nurses by management also creates further divides and resentment among these two cadres. While the GNMs and ANMs in the two facilities did support each other during duty hours (for example, by exchanging shifts if needed), they were reluctant to stand up for each other in relation to the management. Overall, we found that collaborative relationships between ANMs and other facility staff were often missing, and “power with” expressions were muted.

#### Power within

ANMs routinely navigate a power-unequal context and work in a resource-poor setting. In doing this, ANMs undoubtedly display considerable “power within”, with a strong sense of their capacity, awareness of their marginalisation, and a relatively high sense of self-worth. Some of the sense of self-worth of the ANMs in the two facilities studied seemed to stem from their employment in the public sector, which is perceived as prestigious. In-depth interviews with the ANMs revealed that although many found the working conditions difficult, they would never leave the job as they greatly valued the social and financial empowerment it afforded. Not only did it enhance their standing in their own family, but, as one mentioned:

*“We are respected in our communities, we can freely move around in the communities, and people call us didi [sister] and ask for all kinds of information and help.”* (ANM, Facility B)

In a context like Esma, with poor employment opportunities in general (and for women in particular), especially in rural settings, a stable government job is highly valued. Being able to serve the women in their communities through a governmental platform was considered a significant achievement for a woman.

Additionally, some of the “power within” seemed to be rooted in ANMs acquiring new skills and knowledge, and in having the ability to undertake technically more complex work in the labour room. In Facility A, ANMs who served as facility mentors for other ANMs would often state with pride that “we know much more than the doctors here”.

Despite the sense of self-worth, we observed that muted forms of “power to” and “power with” often diminished the true potential of the “power within” ANMs. We found that ANMs were often denied rightful recognition and compensation, even when they acquired advanced clinical skillsets through additional training. The denial of recognition for their enhanced skillsets contributed to their low motivation:

*“We don’t have any promotion. We get a 5% increment irrespective of the work we are doing. We get as much money as an outreach ANM [who is not trained clinically] and the same level of career growth as them. Even if we learn and adopt new practices that are being taught [during additional clinical training], we do not get any career jump from learning anything new. Working in the labour room impacts us negatively as everyone holds us accountable, without appreciating what we do. We were recruited as an ANM and we will retire as one.”* (ANM, Facility B)

Thus, under conditions where their “power to” and “power with” were limited, we found that ANMs could not always recognise and work with their “power within”.

### Expressions of power and quality of care

Our findings suggest that the predominant “power over” expression, along with the limited expression of other forms of power, had many consequences for the quality of service delivery by ANMs in the two facilities studied. Some of these consequences related directly to the lack of appropriate space, toilets, and security. For instance, the lack of access to clean toilets of their own led ANMs to use the unhygienic general and labour room toilets, which contributed to poor infection management practices in the labour rooms. Similarly, the fact that ANMs did not occupy the nursing station because of poor ventilation often led to births in the labour room being left unattended. The absence of adequate security meant that ANMs could not stop family members entering the labour room and distracting them from their work.

Further, we observed that ANMs perpetuated the disrespect they faced in the facilities. We found that ANMs behaved towards the community as the managerial cadres behaved with them, with a perverse “power over” expression dominating their actions. We observed many instances where ANMs asserted a sense of superiority over, and condescension towards, patients, reproducing the patriarchy they were subjected to themselves. They often talked down to women and, when describing patients to us they referred to them as “these women”. ANMs sometimes explained to us how “no matter how much we make them [the community women] understand … poor things, they are illiterate and don’t understand”. There were many instances where ANMs’ interactions with women were disrespectful and abusive, mainly verbally. Sometimes disrespect was also manifested in the way the women were handled physically by the ANMs. The ANMs did not consider their behaviour abusive; they justified their actions by saying that “this is the only language they [the community women] understand”.

Another adverse impact of the predominant “power over”, and the limited expression of other forms of power, related to ANMs’ discretion regarding choosing what work to do. We found that ANMs often coped by intentionally not applying their knowledge in clinical service provision, resulting in “know–do” gaps. This was mainly in response to the unreasonable burden of work placed on them, with little option to negotiate their lack of time, and the burden of doing everything they were asked to do. We observed instances of ANMs being unwilling to sterilise equipment as prescribed, augmenting labour through uterotonics to hasten the birthing process, and avoiding filling in case sheets even when they were able to do so. We found that ANMs also resorted to informally managing their shifts and leave by creating informal rosters. This often led to facilities having even fewer ANMs, unbeknown to management. ANMs routinely accepted petty bribes from patients, and often saw this as justified, since in their view it compensated for their poor remuneration by the system and delays in payment of their salaries. Similarly, to cope with the need to meet the practically unattainable targets set by the district-level officials, ANMs also routinely manipulated labour room registers and documents to over-report their achievements.

Overall, our findings suggest that many of the behaviours that ANMs displayed are not conducive to the provision of good-quality services at the health facilities. The dominant “power over” expression in our findings, largely based on force and domination, formal as well as informal, seems to have undermined the ability of ANMs to provide quality care, as well as demotivating them and leading them to adopt several suboptimal behaviours.

## Discussion

ANMs in Esma are critical to the delivery of sexual, reproductive, maternal, neonatal, and child health services. In this study, we have used the expressions of power framework^24^ to highlight gendered power relations that constrain the performance and quality of care provided by ANMs in the public primary healthcare system in India.

We found that the lack of structural power of the ANMs in the two study facilities, stemming from their position at the bottom of the organisational hierarchy, cascaded across other dimensions of power, limiting their everyday formal and informal power. This, in turn, allowed managers to have almost complete “power over” them. ANMs’ “power to” exercise autonomy and decision-making in their roles, or to challenge existing asymmetrical hierarchies, was found to be very limited. Further, within the health facilities, there were found to be few opportunities for collective action and alliance-building among staff, undermining the “power with” abilities of all cadres. Finally, ANMs’ “power within” was found to be constrained; their agency could only be exercised where this did not challenge the power status quo. Our power analysis also highlighted that the different expressions of power we observed operated in interlinked and interdependent ways, overall resulting in dominant “power over” expressions. Table 4 summarises the study findings.

**Table 4.** Expressions of power

Expression of power	Summarised findings
Power over	Dominant expression of power. The supervisory cadre with authority over ANMs were mostly males, and were not always sensitive to the needs of ANMs. The spaces designated for ANMs in the health facilities were not always available to them, leading to uncomfortable working areas and compromised quality of care. Low levels of security were available to ANMs. ANMs were often the *de facto* providers of sexual, reproductive, maternal, and child healthcare, and hence often bore the brunt of criticisms for poor facility performance. There was an asymmetry between ANMs’ multiple responsibilities and the low levels of formal power conferred on them.
Power to	ANMs had limited ways to exercise their agency with respect to clinical decisions in the health facilities. Shift rosters were often based on doctors’ desires, rather than ANMs’ concerns ANMs were told to “manage somehow” and to deliver the needed service despite infrastructural constraints. ANMs felt they must comply with decisions made by management. ANMs feared punitive actions and reprisals from the facility authorities.
Power with	Collaborative relationships between ANMs and other staff in the facilities were missing. ANMs’ participation in meetings was largely limited to providing work status reports and submitting requests for labour room supplies.
Power within	ANMs’ self-worth seemed to stem from the fact that they were employed in the public sector, which they perceived to be prestigious. Despite the sense of self-worth we observed, muted forms of “power to” and “power with” often diminished the true potential of the “power within” ANMs. The denial of recognition and pay for enhanced skillsets contributed to low motivation. ANMs could only exercise power within in ways that did not challenge the status quo.

Furthermore, we found that the different expressions of power observed were fundamentally entwined with the dynamics of gender. We did not initially adopt a gender lens in conducting the ethnography; however, the structural linkages to the low power positioning of ANMs laid bare the gendered power relations, given that much of the relative power and privilege in the two facilities seemed to lie with managers and medical officers, who were all male. The gender-based biases and discrimination experienced by the ANMs in the study facilities arose due to their weak institutional positioning and were reinforced due to the systemic neglect of ANMs in the state. This neglect is reflected in the lack of a nursing directorate in Esma, limited representation of ANMs and GNMs in policymaking positions in the state, high numbers of supervisory and managerial vacancies, and a lack of policy and regulatory reform related to pre-service education and career progression.^21,33^ This resonates with the more recent literature on deconstructing gender dynamics in health systems.^15,17^

Notions of gendered power hierarchies in health systems are not new; indeed, recent literature has highlighted several gender-related issues in such systems, including the lower social value placed on predominantly female professions, the devalued status and pay-scales of those professions, and a lack of professional networks for them.^6,14,34^ It is increasingly being recognised that gendered relations of power are structurally embedded and have profound impacts on the performance of female healthcare providers.^2,17,34–37^ Our study adds to the empirical literature in this space.

Our study also reveals how extracting services through dominance and control over a disempowered ANM cadre is an injustice not only to them but also to the patients whose service needs and rights to good quality care are devalued in this process. For example, we found that ANMs left births unattended because of uninhabitable nursing stations they had limited “power to” improve. Further, our study findings suggest that the ANMs in the study facilities treated patients with disrespect, perpetuating the disrespect they faced from the managers in those facilities. Disrespectful service provision by facility service providers like ANMs and GNMs in India has been identified as a concern by other studies.^38^ From our findings, we argue that disrespectful service provision by ANMs and GNMs can be considered an outcome of the gender-based neglect of these cadres.

Our findings suggest that cadres such as ANMs need to be empowered in substantive and meaningful ways in order to address inequities and injustices inherent in the provision of services. However, we acknowledge that empowering a cadre is not an easy task. The notion of empowerment has been challenged in the context of community health workers by recognising the fact that such workers have high self-efficacy and exhibit much ingenuity and resourcefulness in undertaking their tasks in under-resourced settings.^7^ However, as our findings suggest, while ANMs exhibit self-efficacy in navigating and negotiating their context, using discretionary power, their positions within the health system cause them to become disempowered. The use of informal and private forms of decision-making to renegotiate power relations without disturbing the structural status quo and gendered culture constitutes only a sub-component of empowerment.^39^ Systemic efforts are needed to empower ANMs. Empowerment strategies, through structural and organisational changes, need to realign gendered power relations and create the “antecedents” to individual empowerment.^40^ The literature notes that even while gendered power dynamics are deeply entrenched, they are also “malleable and subject to change”.^34^ Based on our experience, and learning from the wider literature,^35,41,42^ we put forth below some broad suggestions for shifting from dominant “power over” expressions in relation to ANMs towards more collaborative and equitable relationships.

First, given how other sources of power are constrained by managerial “power over” ANMs, we suggest some ways to move away from such expressions. Literature from other settings highlights the need for managers to be sensitised to issues of power and gender and supported in the process of learning to share power and leadership.^43^ There is also a need to invest in coaching and mentoring supervisors and managers, to make them more supportive. This is an approach that has worked well in the context of community health workers.^44^ Managers in the Esma setting must be particularly sensitised to the routine discriminatory practices that occur in health facilities (such as inequitable access to space for rest and toilets for ANMs), and they must be encouraged to take action. It is also critical that the female cadre of managers mandated in Esma are actually hired and deployed. It is also important that, once managers are sensitised, there are formal mechanisms for holding them accountable for ensuring gender-responsive practices and behaviour in the facilities.

Second, to ensure that ANMs have more “power to” shape their environment, concerted effort should be made to ensure that senior ANM representatives are placed in decision-making positions. Opportunities for nurses and midwives to engage in policymaking with the potential for developing more fair and equitable policies are currently stymied by the state not having a directorate for nursing and midwifery. Even the appointment of nursing tutors to positions equivalent to senior bureaucrats, which is a provision in the state nursing rules, has long been pending. The Nursing Council established at the state level has limited involvement in regulation, and it is headed by a male bureaucrat who lacks deep experience with this cadre – which in this case is entirely female. Further, explicit policies on gender sensitivity (including workplace harassment) should be developed, alongside efforts to ensure that ANMs are provided with adequate infrastructure and resources to enable them to provide quality services. Grievance redressal mechanisms also need to be developed that promote reciprocal accountability of the system to ANMs, not just of ANMs to the system. Although India’s Supreme Court has recommended forming committees related to working condition grievances, many states in India, including Esma, have yet to act on this.^33^

Third, to support ANMs to develop “power with”, interventions to build alliances within and across health facilities should be developed. In other settings, institutionalising peer support and networks,^45^ and investing in appropriate leadership that promotes equitable fora for participatory decision-making^46^ have been tried. Supporting the development of ANM unions or equivalent may also help rebalance power dynamics.

Finally, building “power within” at the individual level will require strengthening pre-service and in-service education. Such education should cover gender awareness and soft skills for persuasion, negotiation, and communication. This recommendation is in line with previous literature that notes the need for imparting counselling skills, and not just technical skills, to frontline health workers,^47,48^

Our study is not without limitations. First, our findings are based on data from two health facilities located in the Indian state of Esma and should be interpreted as such. It would be useful to investigate comparatively the gendered power relations in different health service contexts within India and other LMIC contexts. Second, an explicit gender-focused approach was not adopted at the outset of the study. During the analysis, a grounded theory of gender as a key dimension of power emerged from the data, but this precluded bringing a multi-level and multi-actor perspective to such issues. Finally, some methodological challenges inherent in ethnographic research and framing – especially the issue of reflexivity – should be noted. Equally, the Hawthorne effect, which embedded ethnographic research is meant to counter, can still cause issues in a focused, time-bound ethnographic approach.

We are aware that not naming the state can limit the transparency, understanding, and practical applicability of a study. However, to minimise the limitation this poses, a description of the health system and broader state context within which ANMs operate in Esma is provided to guide readers’ interpretation of the findings. We feel that the paper provides valuable insights on gender-based power asymmetries that are applicable across various states that are resource-constrained and that have poor gender indicators, similar to Esma, and where ANMs are likewise key to sexual and reproductive health provision.^2^ Given that ANMs in Esma were placed similarly to the GNMs in the facilities, our findings also speak to the situations of GNMs, and thus we feel that the study has applicability beyond the ANM cadre. Further, we recognise that deciding not to name the state undermines accountability. However, the situation of ANMs, as reflected in the literature cited in this paper, is not unique to Esma. Hence the evidence provided could support broader advocacy and accountability efforts towards bettering the state of ANMs across India.

## Conclusion

This paper has used the expressions of power framework^24^ to detail how power asymmetries affect the lived experiences and behaviours of ANMs, and how these asymmetries constrain the quality of care they provide. Our findings illustrate how ANMs’ position at the bottom of the official hierarchy allows management and doctors to exercise “power over” them, severely curtailing their legitimate access to and expression of all other forms of power. In the context of ANMs, these expressions of power are fundamentally entwined with dynamics of gender.

The gendered power focus of this paper makes some notable contributions to the literature on the power asymmetries inherent in health systems, and their critical impact on provider performance. The paper demonstrates how “power over” ANMs, as well their limited “power to” control the requisite infrastructural resources necessary for delivering quality care, contributes to the suboptimal provision of care by ANMs. Our findings advance the understanding of gender as a structurally embedded dimension of power and speak to the absence of gender-sensitive policies in health systems.

The paper also suggests some broad directions for empowering ANMs. First, empowering ANMs requires more than skill-building: ANMs must have a formal role in policymaking and decision-making related to their service, and substantive representation in leadership, supervisory, and managerial positions in the health system. There is also a need for policy reforms that enable career progression and the building of support groups. Managers need to be sensitised and coached to provide supportive supervision. In summary, we need to heed the World Health Organization’s^49^ call that “policies to date have attempted to fix women into inequitable systems; now we need to fix the system and work environment”.

## Footnotes

* A minimum qualification of a Bachelor of Science degree in nursing.

† As per personal communication with state-level authorities, the Indian Public Health Standards guidelines developed by the Government of India to improve the quality of the delivery of healthcare do not apply to Esma because of its population density and weak state capacity. For maternal and newborn care, the state authorities adhere to the MNHT of the Government of India. The table presented here relates to the MNHT guidelines for human resources which are expected in the maternity wings of Community Health Centres.

‡ A written request for an explanation of why an employee should not face formal disciplinary action
